# Human *Tsukamurella* infections – Clinical spectrum, diagnosis, antimicrobial resistance, and treatment outcomes: A review

**DOI:** 10.17305/bb.2026.13497

**Published:** 2026-05-15

**Authors:** Bandar Hasan Saleh, Sulaiman Bani Abdel-Rahman, Hala Altarawneh, Noha A Juma, Manal A Zubair, Mona Abdulrahman Alqarni, Ala A Azhari, Nabeel Hussain Alhussainy, Noof R Helmi, Jawahir A Mokhtar, Dalya M Attallah, Malaz Gazzaz, Khulud A Alhazmi, Turki M Alharthi, Asim T Sharif, Bayan Redwan, Khalil K Alkuwaity, Waiel S Halabi, Ahmed Gassim Bukhari, Turki Asiri, Karem Ibrahem

**Affiliations:** 1 Department of Clinical Microbiology and Immunology, Faculty of Medicine, King Abdulaziz University, Jeddah, Saudi Arabia; 2 Department of Clinical Microbiology Laboratory, King Abdulaziz University Hospital, Jeddah, Saudi Arabia; 3 Department of Microbiology and Pathology, Faculty of Medicine, Mutah University, Al-Karak, Jordan; 4 Vaccines and Immunotherapy Unit, King Fahd Medical Research Center, King Abdulaziz University, Jeddah, Saudi Arabia; 5 Pharmaceutical Practices Department, College of Pharmacy, Umm Al-Qura University, Makkah, Saudi Arabia; 6 Department of Microbiology and Parasitology, Faculty of Medicine, Umm Al-Qura University, Makkah, Saudi Arabia; 7 Department of Clinical Laboratory Sciences, Faculty of Applied Medical Sciences, Umm Al-Qura University, Makkah, Saudi Arabia; 8 Department of Medical Education, Faculty of Medicine, King Abdulaziz University, Jeddah, Saudi Arabia; 9 Department of Medical Laboratory Sciences, Faculty of Applied Medical Sciences, King Abdulaziz University, Jeddah, Saudi Arabia; 10 EcoHealth Research Unit, King Fahd Medical Research Center, King Abdulaziz University, Jeddah, Saudi Arabia; 11 Department of Optometry, Faculty of Applied Medical Sciences, University of Jeddah, Jeddah, Saudi Arabia; 12 Department of Microbiology and Parasitology, Faculty of Medicine, King Abdulaziz University, Rabigh, Saudi Arabia

**Keywords:** *Tsukamurella*, opportunistic infections, antimicrobial resistance

## Abstract

*Tsukamurella* species are rare environmental aerobic actinomycetes that are increasingly recognized as opportunistic human pathogens, particularly among immunocompromised patients and those with indwelling medical devices. This review aims to summarize the clinical spectrum, diagnostic methodologies, patterns of antimicrobial resistance, and treatment outcomes associated with human *Tsukamurella* infections. A narrative review of published case reports, case series, and microbiological studies was conducted, emphasizing clinical manifestations, laboratory identification, antimicrobial susceptibility, and therapeutic interventions. The available evidence indicates that catheter-related bloodstream infections are the most frequently reported presentations; however, pulmonary, ocular, cutaneous, endocardial, central nervous system, peritoneal, and prosthetic joint infections have also been documented. Diagnosis remains challenging, as *Tsukamurella* can be misidentified by conventional methods; precise identification often necessitates advanced techniques such as matrix-assisted laser desorption/ionization time-of-flight mass spectrometry (MALDI-TOF MS) and 16S ribosomal ribonucleic acid (16S rRNA) gene sequencing. Successful management typically involves prolonged targeted antimicrobial therapy and the removal of infected devices. In conclusion, *Tsukamurella* is an under-recognized pathogen, and standardized diagnostic and susceptibility-testing protocols, along with larger clinical studies, are essential to enhance patient management.

## Introduction

Taxonomically, *Tsukamurella* is classified within the phylum Actinobacteria, order Actinomycetales, suborder Corynebacterineae, and family Tsukamurellaceae. The genus was first established in 1988 by Collins and colleagues, building upon the earlier work of Japanese microbiologist Michico Tsukamura and studies conducted by Steinhaus [1–3]. *Tsukamurella* species are environmental saprophytes that have been isolated from various sources, including soil, water, arthropods, sludge foam, and marine sponges. While primarily environmental organisms, *Tsukamurella* spp. are recognized as opportunistic pathogens that can be transmitted through contaminated clinical instruments, such as indwelling catheters. In humans, they have been associated with a range of infections, including pulmonary disease, cutaneous lesions, and meningitis, and may colonize immunocompromised individuals without causing overt disease. To date, at least 12 species within the genus *Tsukamurella* have been reported in association with human infections, including *T. inchonensis*, *T. paurometabola*, *T. strandjordae*, *T. tyrosinosolvens*, *T. pulmonis*, *T. hongkongensis*, and *T. sinensis* [3–5].

Initially described as a distinct genus in 1988, *Tsukamurella* belongs to the aerobic actinomycetes and is characterized by very long-chain, unsaturated mycolic acids. These Gram-positive, rod-shaped bacteria are frequently misidentified as *Corynebacterium*, *Rhodococcus*, *Nocardia*, or certain nontuberculous *Mycobacterium* species, making molecular techniques essential for accurate identification. Within the mycolic acid–containing taxa of wall chemotype IV, *Tsukamurella* possesses mycolic acids with 64–78 carbon atoms, compared to *Nocardia*, *Mycobacterium* (60–90), and *Rhodococcus*. The genomic G+C content in *Tsukamurella* typically ranges from 67–68 mol%, whereas *Nocardia* shows 64–72 mol%, *Mycobacterium* 62–70 mol%, and *Rhodococcus* 63–73 mol%, with some exceptions in each genus [1, 3]. Another distinguishing chemotaxonomic marker is the presence of tuberculostearic acid: it occurs in *Tsukamurella*, *Nocardia*, *Mycobacterium*, and *Rhodococcus*, but is generally absent in *Corynebacterium*, aside from certain exceptional species [1, 3].

**Figure 1.**
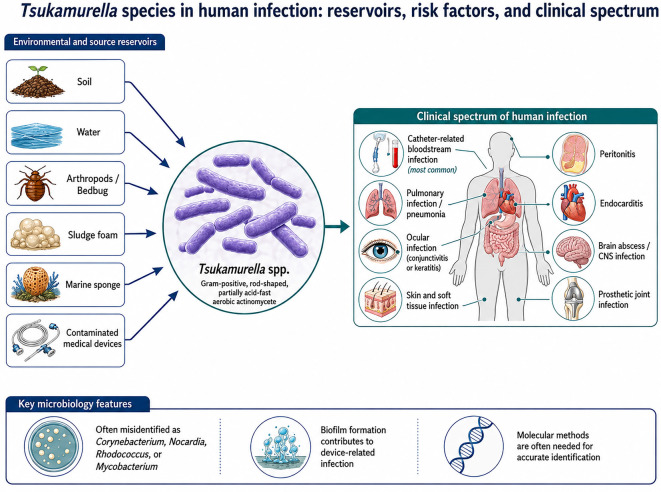
**Environmental reservoirs, microbiological characteristics, and clinical spectrum of human *Tsukamurella* infection.***Tsukamurella* spp. are rare environmental aerobic actinomycetes that may be recovered from diverse ecological reservoirs, including soil, water, arthropods such as bedbugs, sludge foam, marine sponges, and contaminated medical devices. Although primarily saprophytic, these organisms can act as opportunistic human pathogens, particularly in immunocompromised patients and individuals with indwelling medical devices. The most frequently reported clinical manifestation is catheter-related bloodstream infection, while less common presentations include pulmonary infection or pneumonia, ocular infection such as conjunctivitis or keratitis, skin and soft tissue infection, peritonitis, endocarditis, brain abscess or other CNS infection, and prosthetic joint infection. Key microbiological features include Gram-positive, rod-shaped, partially acid-fast, aerobic morphology; frequent misidentification as *Corynebacterium*, *Nocardia*, *Rhodococcus*, or nontuberculous *Mycobacterium*; biofilm formation, particularly in device-related infection; and the need for advanced molecular methods for accurate species-level identification. Abbreviation**:** CNS, central nervous system.

In 1941, Steinhaus [2] isolated an organism from the mycetoma and ovaries of bedbugs (*Cimex lectularius*), naming it *Corynebacterium paurometabolum* [2]. The presence of meso-diaminopimelic acid and an arabinogalactan polymer in its cell wall often led to misidentification, as these strains closely resemble *Corynebacterium* [1, 2]. However, *Tsukamurella* species can be differentiated from *Corynebacterium* by their unsaturated mycolic acids, which contain 68–76 carbon atoms and two to six double bonds [4]. In 1971, Tsukamura and Mizuno described a similar organism with long-chain mycolic acids as *Gordona aurantiaca* [1, 8]. The bacterium later known as *Rhodococcus aurantiacus* was initially classified within the genus *Rhodococcus*, but was subsequently reclassified due to the absence of defining *Rhodococcus* traits. Subsequent studies by Goodfellow et al. demonstrated that *Tsukamurella* shares similarities with *Mycobacterium* and *Nocardia*, yet can be distinguished from other mycolic acid–containing actinomycetes by its very long, unsaturated mycolic acid chains [1, 8] (Figure 1).

Based on 16S rRNA gene sequence analyses, Collins et al. in 1988 reclassified *Corynebacterium paurometabolum* and *Rhodococcus aurantiacus* into the newly established genus *Tsukamurella* [1]. Members of this genus are Gram-positive, partially acid-fast, obligately aerobic bacteria that lack capsules and aerial hyphae. They are chemoorganotrophic, catalase-positive, and pyrazinamidase-positive, with some species capable of producing acid from certain sugars [6]. *Tsukamurella* species are non–spore-forming, lysozyme-resistant, and nonmotile, with rod-shaped cells that may appear straight or curved, either singly, in pairs, or as coccobacillary aggregates under Gram staining [6]. Colony morphology varies, with some species producing pigmentation. Isolation on Löwenstein–Jensen and brain–heart infusion media reveals distinct colony characteristics. During early growth, cells appear as long rods, which later fragment into shorter rods; colonies generally become visible within 24–72 h of incubation [6]. Optimal growth occurs at 25–35 ^∘^C, although exceptions to this temperature range have been reported [9].

The cell envelope of *Tsukamurella* is composed of peptidoglycan, sugars (notably arabinose and galactose, as confirmed by sugar analysis), phospholipids, fatty acids, mycolic acids, and unsaturated menaquinones. Peptidoglycan structural analysis reveals the presence of meso-diaminopimelic acid of type A1. Additionally, the species contains phosphatidylethanolamine and 10-methyloctadecanoic fatty acids. The genomic G+C content typically ranges from 67–68 mol% [10]. The peptidoglycan of *Tsukamurella* consists of *D*-alanine, *L*-alanine, *N*-acetylglucosamine, *D*-glutamic acid, and muramic acid. Its phospholipid profile includes phosphatidylinositol, phosphatidylethanolamine, and diphosphatidylglycerol, while its fatty acid composition features tuberculostearic acid as a characteristic component [3].

*Tsukamurella* spp. are drug-resistant, Gram-positive, aerobic, and partially acid-fast bacteria increasingly recognized as causative agents of bacterial conjunctivitis and keratitis. However, the pathogenesis of *Tsukamurella* keratitis remains poorly understood. Notably, a first-time *in vivo* ocular infection model using New Zealand White rabbits was established, alongside complementary *in vitro* assays to investigate the role of biofilm formation in *Tsukamurella* pathogenesis [11]. Genome sequencing of two ocular isolates—*T. pulmonis* PW1004 and *T. tyrosinosolvens* PW899—was conducted to identify putative virulence factors. Comparative genomic and functional analyses revealed tmytC, encoding a mycolyltransferase, as a key determinant of biofilm formation and virulence. Deletion or inhibition of *tmytC* significantly reduced bacterial adherence, biofilm biomass, and the severity of keratitis in the rabbit model, fulfilling Koch’s postulates for ocular pathogenicity. This report is the first to implicate mycolyltransferase in ocular infection, highlighting its potential as a therapeutic target. Specific inhibitors of TmytC may represent a promising strategy for the management of *Tsukamurella* keratitis and related infections [11] (Table 1).

**Table 1:** Summary of key characteristics of *Tsukamurella*

**Aspect**	**Key highlights (keywords)**	**References**
Taxonomy	Belongs to Actinobacteria, Actinomycetales, Corynebacterineae, Tsukamurellaceae.	[1–3]
Discovery	Genus established in 1988 by Collins based on earlier work by Tsukamura and Steinhaus.	[1–3]
Natural habitat	Environmental saprophyte found in soil, water, arthropods, sludge foam, marine sponges.	[3], [6], [7]
Clinical relevance	Opportunistic pathogen linked to catheter-related infections, pulmonary and cutaneous disease.	[3–5]
Morphology	Gram-positive, rod-shaped, partially acid-fast, aerobic, non-spore-forming bacteria.	[3], [6]
Chemotaxonomy	Contains long-chain mycolic acids (64–78 C) and tuberculostearic acid.	[1], [3]
Misidentification	Often confused with Corynebacterium, Rhodococcus, Nocardia, Mycobacterium.	[1], [3]
Growth features	Colonies visible 24–72 h, optimal growth 25–35 ^∘^C.	[6], [9]
Virulence	Biofilm formation and mycolyltransferase (tmytC) linked to pathogenicity.	[11]

This review aims to comprehensively summarize the clinical manifestations, antimicrobial resistance profiles, and documented treatment outcomes of *Tsukamurella* infections, emphasizing the limited available data and identifying critical areas for future research, including the exploration of phage and antimicrobial peptide-based therapies.

### Epidemiology and pathogenesis

*Tsukamurella* species are rare opportunistic pathogens, predominantly affecting immunocompromised individuals with indwelling central venous access. However, a limited number of cases have also been documented in immunocompetent patients who possess predisposing factors such as end-stage renal disease or uncontrolled diabetes mellitus. Awareness of this organism is essential for timely diagnosis and appropriate management, particularly in high-risk patient populations [12]. This genus is increasingly associated with human infections, with cases reported across Asia, America, Europe, and Africa, indicating its global emergence as a significant pathogen [13].

Human infections caused by *Tsukamurella* spp. are infrequent, with catheter-related bloodstream infection (CR-BSI) being the most commonly documented presentation. Less common manifestations include peritonitis associated with continuous ambulatory peritoneal dialysis (CAPD), respiratory tract infections, skin and soft-tissue infections, brain abscesses, and ocular infections. Greater insight into the clinical and microbiological characteristics of *Tsukamurella* infections is warranted [14–20]. The pathogenesis of *Tsukamurella* remains poorly understood [11].

It has been implicated as a causative agent of infections in immunocompromised individuals, particularly those with indwelling medical devices. Additionally, it has been recovered as a saprophytic organism in a patient with acquired immunodeficiency syndrome (AIDS) [18].

*Tsukamurella* species have long been associated with pulmonary infections, particularly in immunocompromised patients [21, 22]. *Tsukamurella* pneumonia often presents with symptoms resembling those of tuberculosis, which has, in some cases, led to the misdiagnosis of pulmonary tuberculosis in clinical settings [23, 24].

The first documented case of *Tsukamurella* acting as a pathogenic agent in an AIDS patient reported multiple cavitary lung lesions attributable to this organism [18]. Additionally, *Tsukamurella* species have been implicated in a range of opportunistic infections, including meningitis, brain abscesses, prosthetic joint infections (such as knee prosthesis involvement), and cutaneous infections [19, 20, 25, 26].

The largest known case series of *Tsukamurella*-associated ophthalmic infections to date has been documented, focusing on the clinical spectrum, predisposing risk factors, antimicrobial treatment strategies, and patient outcomes. A retrospective review was conducted of all culture-confirmed *Tsukamurella* isolates recovered from ocular microbiological specimens between 2005 and 2018. Identification of *Tsukamurella* species was achieved through a combination of phenotypic characterization, molecular assays, and genotypic sequencing methods. The final diagnosis in each case was based on clinical presentation, supplemented and confirmed by microbiological evidence. Eleven cases of culture-positive *Tsukamurella* ocular infection were identified over the 13-year study period. Conjunctivitis represented the most frequent manifestation, accounting for 54.5% (6/11) of cases. Keratitis was observed in 18% (2/11), and blepharitis in 9% (1/11). Notably, two rare presentations were reported for the first time: one case of canaliculitis and one case of postenucleation ocular implant-related infection. Predisposing factors included the presence of an ocular prosthesis, as well as preexisting ocular surface disorders such as exposure keratopathy and ectropion [27].

Six cases of *Tsukamurella* bacteremia were identified, all occurring in immunosuppressed patients with indwelling central venous catheters (CVCs). All patients achieved full recovery following a combination of targeted antimicrobial therapy and removal of the CVC. These findings underscore the necessity of considering *Tsukamurella* as a potential pathogen in immunocompromised patients with indwelling CVCs who present with Gram-positive bacilli in the bloodstream, particularly when standard identification methods yield ambiguous results [28].

The majority of *Tsukamurella* infections occurred in immunosuppressed patients, often with indwelling CVCs [12]. Notably, a case of CAPD–associated peritonitis caused by *Tsukamurella inchonensis* (*T. inchonensis*) has been documented [29]. Furthermore, a case of catheter-related bacteremia due to *T. pulmonis* was reported in a 39-year-old male patient with acute lymphoblastic leukemia (ALL) undergoing bone marrow transplantation (BMT) [30]. Molecular identification through sequencing of a 1,296 base pair (bp) fragment of the 16S rRNA gene revealed 100% homology with the published sequence of *T. pulmonis* [30]. Additionally, a documented case of community-acquired pneumonia caused by *Tsukamurella pulmonis* was reported [31].

**Table 2:** Summary of the epidemiology, clinical manifestations, and pathogenesis of *Tsukamurella* species infections

**Aspect**	**Key highlights**	**References**
Epidemiology	Rare opportunistic pathogen; mainly affects immunocompromised patients with central venous catheters; globally reported.	[12], [13]
Common infections	Catheter-related bloodstream infection is most common; also peritonitis, pneumonia, skin/soft tissue, ocular infections.	[14–20]
Pulmonary disease	Often mimics tuberculosis and may lead to misdiagnosis.	[22–24]
Risk factors	Immunosuppression, indwelling devices, dialysis, chronic lung disease, diabetes.	[12], [29], [43]
Immunocompetent cases	Rare but reported, including pneumonia and cutaneous infections.	[32–37]
Emerging species	Several novel species identified through molecular methods (e.g., *T. toyonakaense*, *T. sputi*).	[21], [39]
Animal reservoirs	Detected in reptiles and snakes; zoonotic transmission remains unproven.	[41], [42]

**Figure 2.**
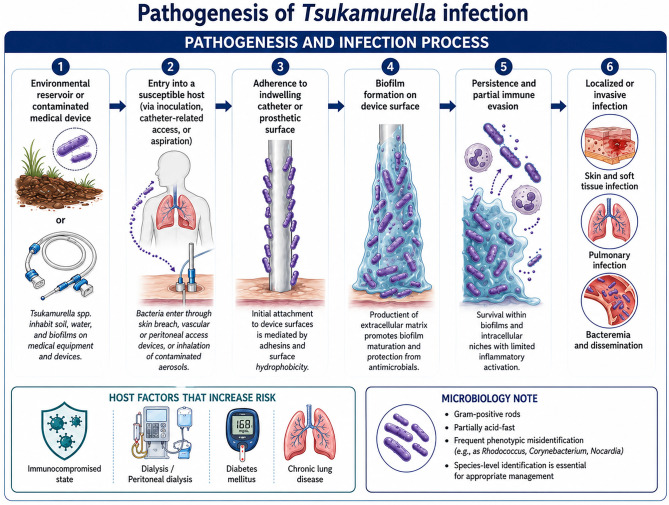
**Pathogenesis of *Tsukamurella* infection.***Tsukamurella* spp. infection may arise after exposure to environmental reservoirs or contaminated medical devices, followed by entry through skin disruption, catheter access, inhalation, or aspiration. After attachment to indwelling catheters, prosthetic material, or damaged tissue, biofilm formation promotes persistence, reduced antimicrobial penetration, and partial immune evasion. Infection may remain localized, causing skin, soft tissue, or pulmonary disease, or progress to bacteremia and dissemination, especially in patients with immunosuppression, dialysis, diabetes mellitus, chronic lung disease, or indwelling devices. Accurate identification is important because *Tsukamurella* spp. are often misidentified as related aerobic actinomycetes.

Recent research indicates that even immunocompetent patients are susceptible to infection. While infections due to *Tsukamurella* species typically occur in immunocompromised individuals, they can, albeit rarely, develop in immunocompetent individuals, sometimes mimicking tuberculosis [32]. Pneumonia was reported in an elderly immunocompetent woman with chronic lung disease caused by *Tsukamurella pulmonis*. Moreover, pneumonia attributed to *Tsukamurella* species has been documented in a 79-year-old immunocompetent patient initially diagnosed with a mycobacterial infection based on the presence of acid-fast bacilli in sputum [33].The infection was confirmed using bacteriological and molecular methods and was treated empirically with antibiotics, highlighting the pathogen’s potential role in pneumonia among immunocompetent patients [34]. Reports also indicate that a respiratory infection caused by *T. tyrosinosolvens* affected a patient without structural lung disease or known immune deficiency [35]. An immunocompetent 24-year-old woman with *Tsukamurella* pneumonia was initially misdiagnosed with pulmonary tuberculosis for nine months. The pathogen was subsequently identified via 16S rRNA sequencing from sputum and bronchoalveolar lavage [22]. Additionally, an unusual case of a mucosal infection caused by *Tsukamurella* species following a nasal septum procedure in an immunocompetent patient has been documented [36]. Interestingly, a 49-year-old immunocompetent woman with a chronic hand lesion, following a suspected spider bite, was successfully treated with prolonged minocycline therapy after culture and tissue analysis identified *Tsukamurella tyrosinosolvens* [37]. Conversely, it has been suggested that in immunocompetent patients, *T. pulmonis*may indicate colonization rather than active infection in respiratory samples [38] (Figure 2).

Clinical samples from patients suffering from respiratory infections, bacteremia, and conjunctivitis revealed the presence of three novel *Tsukamurella* species: *Tsukamurella sputi*, *Tsukamurella asaccharolytica*, and *Tsukamurella conjunctivitidis*. Based on phenotypic characteristics and genomic analyses, including 16S rRNA and housekeeping gene sequencing, they were differentiated from previously known *Tsukamurella* species [39]. A respiratory specimen initially misdiagnosed as nontuberculous mycobacterial pulmonary disease was later recognized as *Tsukamurella toyonakaense* [21]. Through phenotypic and genomic analyses, a new species, *Tsukamurella asaccharolytica*, was identified from a clinical isolate. Sequencing and DNA hybridization confirmed its distinction from other *Tsukamurella* species associated with human infections [39]. Using phenotypic and molecular analyses, it has also been isolated from conjunctival swabs of patients with conjunctivitis. Two novel species were identified, *Tsukamurella ocularis* and *Tsukamurella hominis*, which are closely related to *T. tyrosinosolvens* and *T. pulmonis*, thereby expanding the diversity of ocular-associated *Tsukamurella* species [40].

*Tsukamurella serpentis*, a novel species distinct from other members of the genus *Tsukamurella*, has been isolated from the oral cavity of Chinese snakes and characterized phenotypically and molecularly [41]. *Tsukamurella* species have also been detected in the oral and cloacal samples of captive ball pythons, suggesting that reptiles may serve as environmental reservoirs for transmission [42]. While *Tsukamurella* spp. have been identified in various animals, their role in zoonotic transmission remains unclear and should be regarded as a plausible yet unproven hypothesis that requires further epidemiological and molecular investigations.

**Table 3:** Summary of diagnostic approaches for *Tsukamurella* species

**Diagnostic method**	**Key highlights**	**References**
Phenotypic tests	Primary laboratory approach, but limited for accurate species-level identification.	[3], [41]
Culture characteristics	Growth on blood, chocolate, BHI, Sabouraud, and Löwenstein–Jensen media; colonies are small, dry, convex.	[3], [41], [44]
Microscopy	Gram-positive rods; partially acid-fast staining with weak positivity.	[3], [44]
MALDI-TOF MS	Rapid and reliable method for genus/species identification.	[3], [9], [45], [46]
Molecular methods	16S rRNA sequencing and PCR-based assays used for definitive identification.	[5], [14], [16], [47]
Misidentification	Frequently confused with Rhodococcus, Corynebacterium, Nocardia, or Mycobacterium.	[28]
Advanced techniques	HPLC, DNA–DNA hybridization, and immunological assays support species characterization.	[28], [48]

Abbreviations: 16S rRNA, 16S ribosomal ribonucleic acid; BHI, brain–heart infusion; DNA, deoxyribonucleic acid; HPLC, high-performance liquid chromatography; MALDI-TOF MS, matrix-assisted laser desorption/ionization–time of flight mass spectrometry; PCR, polymerase chain reaction.

**Table 4:** Documented cases of *Tsukamurella* infections: Identification, antimicrobial treatment, and clinical outcomes

**Species**	**Infection type**	**Identification method**	**Treatment**	**Device management**	**Outcome**	**Reference**
*T. pulmonis*	Bacteremia	16S rRNA sequencing	Ceftriaxone (14 days IV)	Not reported	Complete recovery	[12]
*T. inchonensis*	CAPD-associated peritonitis	MALDI-TOF MS + 16S rRNA sequencing	Meropenem	Peritoneal catheter management	Recovery	[29]
*T. tyrosinosolvens*	Chronic otitis media with cerebellar abscess	Culture + molecular identification	Imipenem + amikacin → clarithromycin + TMP-SMX + ciprofloxacin	Surgical debridement	Successful recovery	[20]
*T. pulmonis*	Pleural infection	16S rRNA sequencing	Levofloxacin + rifabutin	Not reported	Clinical improvement	[32]
*Tsukamurella sp.*	Prosthetic joint infection	Culture + molecular identification	Vancomycin + piperacillin/tazobactam → clarithromycin + ciprofloxacin + ethambutol	Implant management	Recovery	[26]
*T. pulmonis* / *T. tyrosinosolvens*	Conjunctivitis	16S rRNA sequencing	Topical polymyxin B–neomycin or chloramphenicol	Not applicable	Resolution within 10 days	[53]
*Tsukamurella sp.*	Endocarditis	Molecular identification	Imipenem + trimethoprim–sulfamethoxazole (6 weeks)	Not reported	Successful outcome	[52]

Abbreviations: 16S rRNA, 16S ribosomal ribonucleic acid; CAPD, continuous ambulatory peritoneal dialysis; IV, intravenous; MALDI-TOF MS, matrix-assisted laser desorption/ionization–time of flight mass spectrometry; TMP-SMX, trimethoprim–sulfamethoxazole.

**Table 5:** Summary of antimicrobial resistance and treatment strategies for *Tsukamurella*

**Aspect**	**Key highlights (keywords)**	**References**
Resistance profile	Resistance commonly reported to penicillin, oxacillin, piperacillin–tazobactam, cephalosporins	[3], [43], [49]
Susceptible antibiotics	Generally susceptible to amikacin, ciprofloxacin, carbapenems, doxycycline, linezolid, sulfamethoxazole	[3], [43], [49]
Genetic resistance	gyrA mutation (S91R) associated with quinolone resistance (levofloxacin, ciprofloxacin)	[24], [43]
Empirical therapy	Often requires combination therapy due to limited treatment guidelines	[18], [28]
Recommended regimen	β-lactam + aminoglycoside, or carbapenem-based combinations with device removal	[18], [28]
Alternative therapy	Fluoroquinolone + rifampin reported as effective oral regimen	[18]
Device-related infections	Removal of infected catheters or implants improves outcomes	[18], [28]
Clinical management	Treatment duration not standardized; depends on infection severity and patient response	[49]
Experimental/Immunostimulatory studies	Heat-killed T. inchonensis improved growth, intestinal morphology, and immune response in broiler chickens and rainbow trout; reduced cholesterol and oxidative stress markers in trout	[54], [55]
Novel therapies	No studies reported on phage or antimicrobial peptide therapy against Tsukamurella	–

Abbreviations: GyrA, DNA gyrase subunit A; S91R, serine-to-arginine substitution at position 91.

A retrospective study collected clinical isolates initially identified as *Tsukamurella* spp., along with corresponding clinical data, from the Clinical Microbiology Laboratory of Peking Union Medical College Hospital in Beijing, China, between 2018 and 2022 [43]. A total of 15 isolates were identified: 12 were associated with probable pulmonary infections in 10 patients, while three were linked to CR-BSI in a single patient. Among the 10 patients with probable pulmonary infections, seven were aged ≥60 years, one was a teenager, and seven were female. Six patients had received steroid therapy, five had underlying immune disorders, and four had chronic lung disease. Bronchiectasis was observed in four patients [43]. Co-infections were identified in six cases, including *Pseudomonas aeruginosa*, *Staphylococcus aureus*, *Enterobacter cloacae*, and *Pneumocystis jirovecii*. Tuberculosis was clinically suspected in five patients, three of whom received prolonged anti-tuberculosis therapy based on negative microbiological findings but a presumptive diagnosis. Notably, one patient (Patient 10) had been diagnosed with tuberculosis in a local hospital after an acid-fast positive sputum smear; however, subsequent cultures were negative for *Mycobacterium tuberculosis* and positive for *T. pulmonis* and *T. inchonensis* [43] (Table 2).

### Diagnosis

The increasing recognition of aerobic actinomycete species presents challenges for clinical microbiologists in achieving accurate identification due to the continual discovery of new taxa. Phenotypic tests remain the primary method for distinguishing aerobic actinomycetes; however, their utility is limited as not all laboratories have the resources for comprehensive species-level identification [3, 41]. For instance, whole-cell sugar analysis is infrequently performed in clinical environments. In medical laboratories, Gram staining is employed to differentiate partially acid-fast organisms from fully acid-fast ones. Most conventional culture media, including blood agar, chocolate agar, brain–heart infusion agar, Sabouraud dextrose agar, and Columbia agar supplemented with 5% defibrinated sheep’s blood, support the growth of these bacteria [3, 41]. Löwenstein–Jensen medium, in conjunction with conventional media, facilitates the growth of most aerobic actinomycetes, with aerobic conditions recommended for optimal growth. *Tsukamurella* species, when subjected to partially acid-fast staining, exhibit weakly positive results. On conventional media, colonies appear small, dry, and convex, with colors ranging from white and cream to orange [44]. The colonies of *Tsukamurella paurometabola* are typically smooth, creamy, and exhibit a distinctive ”fried-egg” appearance, whereas other species may exhibit a spectrum of colors. As noted by Steinhaus, a semisolid medium containing gelatin, carbohydrates, and rabbit serum serves as the foundational medium for isolating *T. paurometabola* [2, 3]. While the species are generally partially acid-fast, some researchers have reported strongly acid-fast results in certain species. Matrix-assisted laser desorption/ionization-time of flight mass spectrometry (MALDI-TOF MS) can also be utilized for accurate identification [3, 9, 45, 46].

*Tsukamurella* species share numerous phenotypic characteristics with related genera, including *Rhodococcus*, *Corynebacterium*, *Nocardia*, and *Mycobacterium*. Due to these similarities, precise identification necessitates advanced molecular techniques, such as polymerase chain reaction-restriction fragment length polymorphism (PCR-RFLP) analysis of the heat shock protein gene (*hsp65*) and 16S rRNA gene sequencing, to reliably differentiate *Tsukamurella* from closely related taxa [5, 14, 16].

**Figure 3.**
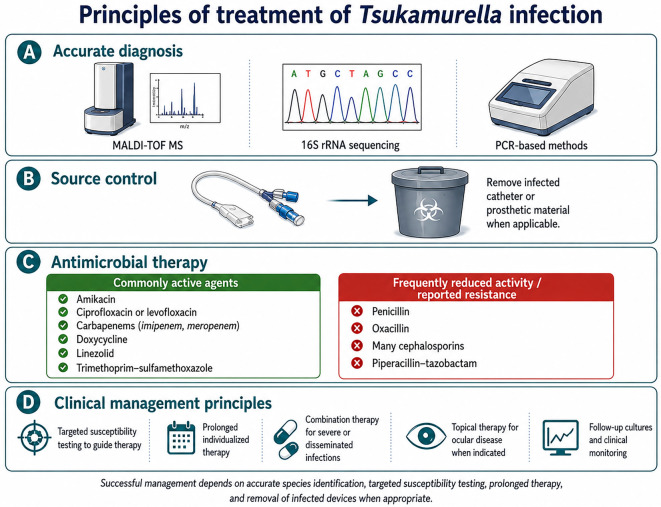
**Principles of treatment of *Tsukamurella* infection.** Management of *Tsukamurella* spp. infection requires accurate species-level diagnosis, preferably using MALDI-TOF MS, 16S rRNA sequencing, or PCR-based methods, because conventional phenotypic testing may lead to misidentification. Treatment should include source control, particularly removal of infected catheters or prosthetic material when feasible, together with targeted antimicrobial therapy guided by susceptibility testing. Reported active agents include amikacin, fluoroquinolones, carbapenems, doxycycline, linezolid, and trimethoprim–sulfamethoxazole, whereas penicillins, oxacillin, many cephalosporins, and piperacillin–tazobactam often show reduced activity or resistance. Prolonged individualized therapy, combination treatment for severe or disseminated disease, topical therapy for ocular infection, and follow-up cultures are key clinical management principles. Abbreviations**:** 16S rRNA, 16S ribosomal ribonucleic acid; MALDI-TOF MS, matrix-assisted laser desorption/ionization time-of-flight mass spectrometry; PCR, polymerase chain reaction.

A patient presented with cloudy peritoneal dialysate, indicative of peritonitis. Culture of the peritoneal fluid yielded yellow-gray, dry, membrane-like colonies. Gram staining revealed straight, Gram-positive rods [29]. The isolate was initially identified as a *Tsukamurella* species using MALDI-TOF MS, and 16S rRNA gene sequencing confirmed close homology with *T. inchonensis* via GenBank database comparison [29].

Molecular identification through sequencing of a 1,296 base pair fragment of the 16S rRNA gene revealed 100% homology with the published sequence of *T. pulmonis* [30].

Diagnostic misidentification is prevalent, as *Tsukamurella* species often resemble *Rhodococcus* or *Corynebacterium* in routine laboratory testing [28].

The utility of high-performance liquid chromatography (HPLC) for genus-level identification was demonstrated in one study, while 16S ribosomal RNA gene sequencing and DNA–DNA hybridization dot-blot assays enabled species-level characterization. Three isolates were identified as *T. pulmonis*, one as *T. tyrosinosolvens*, and one as a previously undescribed species. A sixth isolate was not preserved long enough for definitive identification [28]. Notably, a TaqMan real-time PCR assay targeting a 157-bp segment of the 16S rRNA gene was developed as a rapid and reliable diagnostic tool to detect *Tsukamurella* among 12 strains and 11 related species [47]. Additionally, polysaccharides on the surface of *T. pulmonis* can serve diagnostic purposes and facilitate the study of host-pathogen interactions. Structural characterization of the cell wall polysaccharide through sugar and methylation analyses, alongside two-dimensional nuclear magnetic resonance (NMR), revealed it to be an arabinomannan composed of branched tetrasaccharides with an additional linear →6)-α-D-Manp-(1→ mannan chain. Immunological studies demonstrated cross-reactivity between *T. pulmonis* and *T. paurometabola* using rabbit polyclonal sera. Immunohistochemical staining with monoclonal antibodies against the *T. pulmonis* polysaccharide revealed pathological granules and liver abscesses within cells. Ultrastructural analysis indicated that these granules contained *T. pulmonis* cells, suggesting dissemination of the organism within the host through the bloodstream [48] (Table 3).

### Resistance and treatment

The pathogenic mechanisms and antibiotic resistance of *Tsukamurella* remain poorly understood, underscoring the need for further research utilizing clinical samples and novel detection methods [3]. Notably, *T. inchonensis* exhibits resistance to cephalosporins and variable susceptibility to vancomycin, which may result in the failure of standard empiric antibiotic regimens. These findings highlight the necessity for increased clinical awareness and tailored antimicrobial therapy. Further studies and case analyses are essential to better characterize the clinical behavior of *T. inchonensis* and to establish optimal treatment guidelines for infections caused by this emerging pathogen [29].

According to a multiple-sequence alignment with the reference sequence of Mycobacterium tuberculosis H37Rv (NP_214520.1), a quinolone-resistance-determining region (QRDR) of the gyrA gene in *Tsukamurella* species has been proposed to encompass amino acids 75–114 [24]. However, this region has not yet been experimentally identified in *Tsukamurella* species but is predicted based on cross-genus homology. A unique S91R mutation within the QRDR was detected in the quinolone-resistant isolate 22TM00764, suggesting that this DNA gyrase subunit substitution may contribute to high-level resistance to levofloxacin and ciprofloxacin, while conferring only intermediate resistance to moxifloxacin [43].

To date, a universally accepted method for antimicrobial susceptibility testing of *Tsukamurella* species has not been established. Consequently, the optimal therapeutic strategies for managing *Tsukamurella* infections remain undefined [49]. Severe outcomes of *T. tyrosinosolvens* infection have been documented in immunocompromised patients, including life-threatening events associated with systemic spread. The optimal duration of treatment for *Tsukamurella* infections remains to be determined and should be tailored according to the patient’s clinical response [50, 51]. Notably, *Tsukamurella* isolates typically exhibit susceptibility to amikacin, ciprofloxacin, imipenem, doxycycline, linezolid, and sulfamethoxazole, while demonstrating resistance to penicillin, oxacillin, piperacillin–tazobactam, and cephalosporins [3, 43, 49].

Due to the scarcity of reported cases, the optimal management of *Tsukamurella*-related infections remains ambiguous. Current evidence indicates that a combination of a β-lactam antibiotic and an aminoglycoside, along with the removal of infected foreign bodies, is the preferred treatment strategy [18]. Drawing from treatment protocols for nocardiosis and atypical mycobacterial infections, combination therapy is frequently recommended for *Tsukamurella* infections. The use of a beta-lactam (including carbapenems) in conjunction with an aminoglycoside or a rifamycin, along with the removal of any infected medical devices, appears to represent the most effective approach [28].

Additionally, a successful oral antimicrobial regimen consisting of a fluoroquinolone combined with rifampin has been proposed as an alternative therapeutic option for infections caused by this uncommon pathogen [18].

Standard management for *Tsukamurella*-related conjunctivitis, keratitis, and blepharitis typically involves combination topical therapy using two antibiotics—most commonly a fluoroquinolone alongside fusidic acid or chloramphenicol—resulting in satisfactory clinical resolution. More complex infections, such as canaliculitis and ocular implant-related infections, necessitate escalation of therapy with systemic antibiotics (macrolide or doxycycline), surgical intervention (canaliculotomy), or removal of the infected implant to achieve a cure [27].

A recent case of *Tsukamurella* bacteremia was reported, in which the patient was successfully treated with a 14-day course of daily intravenous ceftriaxone and experienced complete resolution of bacteremia upon discharge [12] (Figure 3).

Antimicrobial susceptibility testing of *Tsukamurella inchonensis* demonstrated sensitivity to quinolones, carbapenems, and linezolid, with intermediate resistance to vancomycin. The patient was effectively treated with meropenem and subsequently discharged [29]. A case of chronic otitis media complicated by a cerebellar abscess and intracranial invasion caused by *Tsukamurella tyrosinosolvens* was documented. The patient was initially treated with intravenous imipenem and amikacin, followed by oral clarithromycin, trimethoprim-sulfamethoxazole, and ciprofloxacin. A successful outcome was achieved through a combination of surgical debridement and prolonged antibiotic therapy [20]. Another case report detailed a 42-year-old man initially misdiagnosed with tuberculosis, later confirmed to have a *Tsukamurella pulmonis* pleural infection, and successfully treated with levofloxacin and rifabutin, resulting in stable recovery [32]. Furthermore, the first case of *Tsukamurella* endocarditis was successfully treated with a six-week course of imipenem and trimethoprim-sulfamethoxazole [52]. Notably, a 69-year-old woman with recurrent postoperative knee wound infections was diagnosed with a *Tsukamurella* sp. infection. She was successfully treated with vancomycin, piperacillin/tazobactam, followed by clarithromycin, ciprofloxacin, and ethambutol, marking the first reported *Tsukamurella* infection of an artificial joint [26]. The first three cases of *Tsukamurella* conjunctivitis were reported; 16S rRNA sequencing identified one isolate as *Tsukamurella pulmonis* and the other two as *Tsukamurella tyrosinosolvens*. All three patients responded promptly to eyedrops containing polymyxin B-neomycin or chloramphenicol. Each patient exhibited unilateral eye congestion with mild serous discharge and recovered after 10 days of treatment [53] (Table 4).

In broiler chickens, heat-killed *Tsukamurella inchonensis* was evaluated. Although it did not affect feed intake or conversion, pulsing improved weight gain (enhancing intestinal morphology), and significantly increased antibody responses, indicating immunostimulatory benefits [54]. Additionally, a study found that heat-killed *Tsukamurella inchonensis* improved growth, intestinal structure, and immune function in rainbow trout while reducing cholesterol and oxidative stress markers without altering key liver enzymes [55]. To date, no studies have reported the use of phage or antimicrobial peptide therapy against *Tsukamurella* species (Table 5).

## Discussion

In immunocompromised patients, *Tsukamurella* species can cause a variety of infections due to their opportunistic nature. Reported infections include pulmonary infections, cutaneous lesions, ocular infections, endocarditis, and infections of prosthetic joints. Despite increasing documentation from various regions, *Tsukamurella* species remain rare and under-recognized pathogens. Their infections may be misdiagnosed as tuberculosis or other Gram-positive bacterial infections due to overlapping clinical features, potentially delaying appropriate treatment. The absence of standardized diagnostic methods and guidelines for susceptibility testing further complicates clinical management.

The pathogenic mechanisms of *Tsukamurella* remain poorly understood. Most findings regarding biofilm formation, virulence factors, and antibiotic resistance stem from case reports and small case series. Consequently, conclusions regarding pathogenesis and optimal treatment strategies should be drawn with caution. Future studies employing genomic analysis, molecular characterization, and experimental infection models are needed to elucidate these mechanisms and identify potential therapeutic targets.

Current literature indicates a scarcity of studies investigating novel therapeutic strategies such as phage therapy or antimicrobial peptides; however, given the limited scope of published data, this observation should be interpreted cautiously. The development of innovative treatment approaches may provide additional options for managing complex or device-associated infections in the future.

This review has several limitations. Drawing definitive conclusions is challenging due to the limited evidence base, which is primarily derived from small observational studies and isolated case reports. Moreover, variations in diagnostic methods and report formats across studies hinder data synthesis. Despite these challenges, the review underscores significant knowledge gaps and emphasizes the need for ongoing multicenter surveillance, standardized diagnostic approaches, and both laboratory and clinical research to enhance our understanding and management of *Tsukamurella* infections.

## Conclusion

Patients with indwelling medical devices and immunocompromised individuals are at a heightened risk of acquiring infections from *Tsukamurella* species, which are rare opportunistic pathogens. The transmission dynamics of these infections, their pathophysiology, and effective management strategies remain inadequately defined. The lack of definitive clinical guidelines stems from the predominance of isolated case reports and small case series in the available evidence. Advancing our understanding of these infections and developing evidence-based treatment strategies will require further multicenter clinical studies, standardized diagnostic methodologies, and molecular investigations.

**Conflict of interest**: Authors declare no conflicts of interest.

**Funding:** Authors received no specific funding for this work.
